# Abuse and its associated factors among elderly population of Kamalamai Municipality of Sindhuli District, Nepal

**DOI:** 10.1371/journal.pone.0316078

**Published:** 2025-01-24

**Authors:** Jwala Subedi, Deepak Kumar Yadav, Paras Kumar Pokharel, Samyog Uprety, Birendra Kumar Yadav, Anisha K. C., Sheetal Bhandari, Samiksha Baral

**Affiliations:** 1 B. P Koirala Institute of Health Sciences, School of Public Health and Community Medicine, Dharan, Nepal; 2 Central Department of Public Health, Institute of Medicine, Tribhuvan University, Kathmandu, Nepal; Global Center for research and Development (GCRD), UNITED STATES OF AMERICA

## Abstract

**Background:**

The global rise in the elderly population brings attention to the pressing issue of elder abuse, categorized into physical, psychological, neglect, financial and sexual abuse. According to the World Health Organization (2022), one in six individuals aged 60 and older has experienced some form of abuse in community setting necessitating increase in awareness and support for older people. This study aimed to assess abuse and its associated factors among elderly population of Kamalamai Municipality of Sindhuli District, Nepal.

**Methodology:**

We conducted a community-based cross-sectional study utilizing both qualitative and quantitative methods. We collected data from June 15 to September 15, 2023. The study involved 398 elderly participants (≥60 years). Stratified sampling techniques was used to select participants. For the qualitative study we utilized purposive sampling technique which included two key informant interviews, one in-depth interview and two focus group discussions. We analyzed quantitative data using IBM SPSS version 20 applying bivariate and multivariable logistic regression analysis at a 95% confidence interval. For qualitative data, thematic analysis was done.

**Results:**

The mean ± SD age of the elderly participants was (70.8± 8.08) years. Among the total of 398 study participants, (64.1%) participants reported experiencing different forms of abuse. The study found that neglect (46.0%), psychological abuse (42.5%), and financial abuse (24.4%) were the most common while physical abuse (4.8%) and sexual abuse (2.8%) were less common. Living in a nuclear family (AOR: 2.74, 95% CI: 1.67–4.51) and lower socio-economic status (AOR: 5.42, 95% CI: 2.28–12.92) were significantly associated with increase risk of elder abuse. Qualitative findings revealed mistreatment, vulnerability, negligence, economic factors, family dynamics, loneliness, financial exploitation, food withholding, physical abuse, and emotional attachment as key aspects of elder abuse.

**Conclusion:**

This study highlights neglect and psychological abuse were the most common forms of elder abuse. These findings emphasize the urgency of implementing programs at the municipal level such as financial support system for elderly individuals from lower socioeconomic backgrounds and education programs for both elderly individuals and their families and caregivers. These initiatives aim to control and reduce the incidence of elder abuse and ensure comprehensive well-being of elderly population.

## Introduction

Ageing is a lifelong process of progressive change in biological, psychological and social structures of a person. It is a common, natural and continuing process. Most developed countries accept the age of 65 as a definition of elderly, but developing countries adopt less of that age. The Government of Nepal has declared that people aged 60 years or older are elderly citizens [1–3].

The World Health Organization (WHO) defines elder abuse as ’a single or repeated act, or lack of appropriate action, occurring within any relationship where there is an expectation of trust, which causes harm or distress to an older person.’ This type of violence constitutes a violation of human rights and includes physical, sexual, psychological, and emotional abuse; financial and material abuse; abandonment; neglect; and a serious loss of dignity and respect [4]. Elder abuse is a global public health issues that significantly increases the risks of institutionalization, hospitalization, and psychological issues such as depression and anxiety [5].

Abuse can happen to anyone, regardless of their age, gender, race, religion, or ethnic and cultural background. The types of abuse defined by the National Institute on Aging (NIA) include physical abuse, psychological abuse, neglect, financial abuse, and sexual abuse which aligns with the types provided by the WHO [6]. This study also assessed these five types of abuse. According to the United Nations, globally there were 703 million individuals aged 65 years or older in 2019,with projections suggesting this number will exceed 1.5 billion by 2050 [7]. WHO reports 1 in 6 individuals aged 60 and older experienced some form of abuse in community settings. The rates of abuse in elderly are even higher in institutions like nursing homes and long-term care facilities. The incidence of abuse against older adults has increased during the COVID-19 pandemic, and with the elderly population steadily increasing globally, cases of elder abuse are expected to rise further [4].

According to the Senior Citizens Act of Nepal (2063) defines senior citizens as individuals who are 60 years and above. According to the 2021 population census, 10.21% of Nepal’s population is 60 years and older, marking a significant increase from 8.13% in 2011. The percentage is projected to increase in the coming decades [8, 9].

According to the various study conducted from 2012 to 2021 revealed alarming cases of elder abuse in Nepal. During this period a total of 2,447 reported cases of abuse were reported, including physical, financial, psychological, sexual, and neglect-related abuse. Furthermore the gravity of the situation becomes more pronounced as the data shows 2,058 elderly individuals lost their lives due to abuse during the same time frame [10].

Elder abuse is an increasing concern in Nepal, that requires urgent government attention due to factors like physical vulnerability and shifting societal norms which make older individuals more susceptible to maltreatment. Increasing longevity and changing societal values have elevated the risk of elder abuse. Although the government’s social security initiatives play a vital role in prevention, current policies are inadequate to address the growing needs of the elderly population [11].

While elder abuse has been studied in various contexts, there is a significant gap in understanding the prevalence, characteristics, and associated factors in developing countries like Nepal [11]. Addressing this gap is crucial for developing strategies and interventions aimed at preventing and addressing elder abuse. The findings from this study will ultimately contribute to enhancing the wellbeing and quality of life of elder population in Nepal. Therefore, this study aimed to assess the prevalence and characteristics of elder abuse as well as its associated factors among the elderly population.

## Materials and methods

### Study design

A cross-sectional study, using mixed methods with a convergent parallel design, was conducted. We collected data from June 15 to September 15, 2023.

### Study population

The study included individuals aged 60 years and older residing in the Kamalamai Municipality of Sindhuli District. The inclusion criteria were elderly individuals who were willing to participate and able to provide informed written consent. From each household, only one elderly individual was selected with preference given to oldest member in cases where multiple elderly members were present.

### Study setting

The study was conducted through a house-to-house survey among elderly residing in Kamalamai Municipality of Sindhuli District. Kamalamai municipality is located in Sindhuli district within Bagmati Province of Nepal, consisting a total of 14 wards. According to the 2021 Census by the Central Bureau of Statistics(CBS), the municipality has a total population of 65,064 individuals, with 7,168 individuals aged 60 and above [12].

### Sampling technique

For the quantitative study, a stratified sampling technique was employed. Among the 14 wards in the municipality, seven wards were randomly selected using a lottery method. Participants were stratified into three age group (60–69, 70–79, and ≥80). Samples were selected from each stratum using population-proportionate sampling technique. The first samples was selected randomly using a lottery method in each ward. Then the remaining samples were taken consecutively until the required number of samples.

For the qualitative study, the purposive sampling technique was used.

### Calculation of the sample size

In quantitative study, sample size was calculated using a single proportion for finite population formula $\text{n}=\frac{Z^{2}pqN}{d^{2}\left(N-1\right)+z^{2}pq}$ [13]. The total number of elderly individuals aged 60 and above in Kamalamai Municipality of Sindhuli district was 7,168 [12].

A proportion of 0.545 was taken from a previous study conducted by Acharya et al. (2021) in Syangja, Nepal [14]. Assuming a 95% confidence interval (CI), a 5% allowable error, and a 10% non-response rate, the final sample size determined as 398 for this study.

### Data collection tools

For the quantitative study, the tool was adapted from Acharya et al. (2021). It was validated in Nepal and has a Cronbach’s Alpha of 0.71, indicating good internal consistency. The questionnaire comprised four distinct parts and variables collected are outlined in the table below Table 1.

**Table 1:** Variables and description on the study.

Variables	Description
Elderly Population	Individuals aged 60 years or older were classified as elderly.
Socio-economic status	The socioeconomic status was assessed using the Kuppuswamy scale This scale considers the education of the head of the family, the occupation of the head of the family, and the total family income per month. The cumulative score on the Kuppuswamy scale ranges from 3 to 29. This scale classifies families into five distinct groups: upper class, upper middle class, lower middle class, upper lower class, and lower socioeconomic class. A score of less than 5 is considered lower class, 5–10 is considered upper lower class, 11–15 is considered lower middle class, 16–25 is considered upper middle class, and 26–29 is considered upper class [15].
Physical Abuse	Utilization of physical force leading to physical pain or impairment, encompassing actions such as hitting, pushing, beating, and burning.
Psychological or emotional abuse	Harassing, using slang words, humiliating, or using gesture language. For instance, controlling behaviours, name-calling, confinement, and isolation, etc.
Neglect	Situations where elderly individuals were left alone, isolated, or forgotten, often involving the withholding of essential daily necessities. This encompassed instances like failure to provide food, housing, or necessary medical care.
Financial Abuse	Financial abuse as the inappropriate use of property or money, such as coercing an older person to sign documents against their will.
Sexual abuse	Any form of sexual contact without consent, which included unwanted physical contact, instances of rape, and the non-consensual photographing of explicit content.

### Data collection techniques

For the quantitative study, face-to-face interviews were conducted using a structured and validated questionnaire in Nepali language. To ensure comfort and honesty of participants response, interviews took place in a private setting, free from distractions. We built a rapport with participants before the formal questioning and assure them that their response would remain confidential making an environment comfort for open communication.

For the qualitative study, two key informant interviews and one in-depth interview were carried out, along with two focus group discussions. The participants were purposively selected: for key informant interviews, the Deputy Mayor and the Head of the Social Development Department of Kamalamai Municipal Office; for the in-depth interview, a general member of *Jestha Nagarik Sindhuli Sangh* (Senior Citizen Association Sindhuli) was chosen. For the focus group discussions, elderly individuals from ward numbers 9 and 13 were selected. The FGD were conducted in areas where we observed the highest level of abuse during the quantitative data collection. The interview and focus group discussion were conducted until data saturation had been reached. The Focus Group Discussions (FDG) Key Informant Interview (KII) and In-Depth Interviews (IDIs) were conducted in Nepali language by following the semi-structured guidelines and the interviews, were tape-recorded.

The data collectors were principal investigator and public health graduates. Two days of training were provided to them on data collection, study tools administration, data handling procedures and ethical aspects of the study before data collection.

### Validity and reliability

The study tools were translated into Nepali language and pretested among 10% of the study sample (n = 40) in Dudhauli municipality of Sindhuli district, which was not included in the final analysis. The result showed Cronbach’s alpha coefficient of 0.74, which fell within an acceptable range, signifying good internal consistency.

The FGD, IDI and KII guidelines were developed under the close guidance of the experts and based on an extensive literature review.

### Ethical approval

Ethical approval was obtained from the Institutional Review Committee (IRC) of B.P Koirala Institute of Health and Sciences **(Ref 688/079/080])** and a letter of support was obtained from Kamalamai Municipality. Informed written consent was obtained from each study participants before the interview was conducted. The study participants were informed about voluntary participation, their right to withdraw from the interview at any point and confidentiality of the data was maintained.

### Data management and analysis

For quantitative study, collected data were entered, filtered, coded, cross checked and exported to IBM Statistical Package for Social Sciences (SPSS) version 20.0 for statistical analysis. Descriptive analysis was conducted using a frequencies, percentages, mean and standard deviations. Inferential analysis was conducted to identify associations between the independent variables and abuse. The Kolmogorov–Smirnov test and Shapiro–Wilk test were performed to assess whether the data followed a normal distribution. Bivariate analysis was performed using the chi-square test to identify predictors associated with abuse. Crude and adjusted odds ratios were calculated to find the strength of association between variables at 95% confidence interval. Variables found significant and p value with less than 0.2 in bivariate analysis was further processed into multiple logistic regression to find the relationship between each variable. Additionally, variables with cell counts of less than 5 were not included in the multivariable analysis. All the statistically test was performed at 95% Confidence Interval and p-value less than 0.05 was considered significant.

Foe qualitative data, an inductive thematic approach was used for data analysis, focusing on identifying recurring ideas, patterns, and concepts that naturally emerged from participants’ discussions without imposing predetermined categories. The coding process was conducted manually, which led to the generation of themes and corresponding subthemes.

## Results

### Result of quantitative study

#### Socio-demographic characteristics of participants

Table 2 Among the 398 participants, the majority 220(55.3%) were in the 60–69 years age group, with males representing 239(60.1%) of the total sample. More than half of the participants 206(51.8%) were Brahmin/Chhetri, while 347 (87.2%) followed Hinduism. Half of the participants 199(50.0%) came from a joint family. The majority of participants belonged to the upper -lower socio-economic status159 (39.9%).

**Table 2:** Socio demographic characteristics of the participants.

Characteristics	Frequency (n)	Percentage (%)
**Age in years**		
**Mean ± SD**	**70.88 ± 8.08**	
60–69	220	55.3
70–79	123	30.9
≥ 80	55	13.8
**Gender**		
Male	239	60.1
Female	159	39.9
**Ethnicity**		
Brahmin/Chettri	206	51.8
Janajati	139	34.9
Dalits	44	11.1
Madhesi	9	2.3
**Religion**		
Hindu	347	87.2
Buddhist	41	10.3
Christian	10	2.5
**Family type**		
Nuclear	155	38.9
Joint	199	50.0
Extended	44	11.1
**Number of family members**		
**Mean ± SD**	**5.64 ± 2.89**	
**Education status**		
Illiterate	332	83.4
Literate	66	16.6
**Marital status**		
Living with spouse	252	63.3
Widow	114	28.6
Widower	32	8.0
**Housing condition**		
Own	371	93.2
Rented	27	6.8
**Occupation**		
Unemployed	141	35.4
Agriculture	126	31.7
Home maker	63	15.8
Retired	37	9.3
Others (shopkeeper, daily wages, land dealer, rent from home)	31	7.8
**Number of children**		
1–3	158	39.7
4–6	192	48.2
≥ 7	43	10.8
Do not have any children	5	1.3
**Living arrangements**		
With family members	362	91.0
Alone	33	8.3
Others (known person)	3	0.7
**Present source of income**		
Yes	339	85.2
No	59	14.8
**Socio-economic status**		
Upper	0	0
Upper Middle	60	15.1
Lower Middle	90	22.6
Upper Lower	159	39.9
Lower	89	22.4
**Income per month**	**(Mean ± SD) (10014.44 ± 17674.09)**	

#### Behavior and health related characteristics of participants

About 179(45.0%) had a habit of using tobacco, while 114(28.6%) consumed alcohol. Regarding health problems, 281(70.6%) of the participants reported experiencing some form of illness (Table 3).

**Table 3:** Behavioral and health-related characteristics of participants.

Characteristics	Frequency (n)	Percentage (%)
**Current habit of Tobacco consumption**		
Yes	179	45.0
No	219	55.0
**Current habit of alcoholism**		
Yes	114	28.6
No	284	71.4
**Suffered from disease in previous six months**		
Yes	281	70.6
No	117	29.4

Table 4 The distribution of abuse types among participants is shown. Out of 398 elderly participants,255 (64.1%) reported experiencing some form of abuse, while 143(35.9%) reported no abuse. Among those who experienced abuse, 102(25.6%) reported only one type,93 (23.4%) experienced two types, 50(12.6%) experienced three types, 9(2.3%) experienced four types, and 1(0.2%) experienced all types of abuse.

**Table 4:** Distribution of abuse types among participants.

Characteristics	Frequency (n)	Percentage (%)
**Only one**	102	25.6
**Two types**	93	23.4
**Three types**	50	12.6
**Four types**	9	2.3
**All types**	1	0.2
**Overall abuse**	255	64.1
**No any abuse**	143	35.9

#### Types of abuse among participants

Table 5 the prevalence of various types of abuse among the participants is shown, with 19(4.8%) experiencing physical abuse,169 (42.5%) suffering from psychological abuse,183 (46.0%) facing neglect,97 (24.4%) reporting financial abuse and 11 (2.8%) facing sexual abuse.

**Table 5:** Prevalence of types of abuse among participants.

Characteristics	Frequency (n)	Percentage (%)
**Physical abuse**		
Yes	19	4.8
No	379	95.2
**Psychological abuse**		
Yes	169	42.5
No	229	57.5
**Neglect**		
Yes	183	46.0
No	215	54.0
**Financial abuse**		
Yes	97	24.4
No	301	75.6
**Sexual Abuse**		
Yes	11	2.8
No	387	97.2

#### Bivariate and multivariable logistic regression analysis

Multivariable logistic regression was conducted for variables such as age, sex, marital status, family type, education status, socio economic status and tobacco consumption habit, whose p-values were less than 0.2.

In Multivariable logistic regression, family types and socio-economic status were found to be statistically significant factors associated with abuse.

This indicates that individuals’ family types are associated with 2.74 times higher odds of experiencing abuse.

Likewise, the analysis demonstrates a significant relationship between socioeconomic factors and the occurrence of abuse. The results indicate that individuals with a lower socioeconomic status are more prone to experiencing abuse when compared to those with higher socioeconomic status (Table 6).

**Table 6:** Independent factors associated with abuse.

Characteristics	Abuse		p-value	COR	AOR
	Yes n(%)	No n(%)			
**Age group in years**					
60–69	145 (65.9)	75 (34.1)	**0.168** ^a^	Ref.	
70–79	81 (65.9)	42 (34.1)		0.99 (0.62–1.58)	0.898(0.53–1.50)
≥80	29 (52.7)	26 (47.3)		0.57(0.31–1.04)	0.528(0.27–1.02)
**Sex**					
Male	146 (61.1)	93 (38.9)	**0.128** ^a^	Ref.	
Female	109(68.6)	50 (31.4)		1.38 (0.90–2.12)	1.24 (0.69–2.22)
**Ethnicity**					
Brahmin-Chettri	126(61.2)	80(38.8)	0.211 ^a^	Ref.	
Non-Brahmin-Chettri	129(67.2)	63(32.8)		1.30(0.86–1.96)	
**Religion**					
Hindu	222(64.0)	125(36.0)	0.919 ^a^	Ref.	
Non-Hindu	33 (64.7)	18(35.3)		1.03(0.55–1.90)	
**Education**					
Literate	33 (50.0)	33 (50.0)	**0.009** ^a^	Ref.	
Illiterate	222 (66.9)	110 (33.1)		2.01(1.18–3.44)	1.19(0.61–2.35)
**Types of family**					
Non-nuclear	131(53.9)	112(46.1)	**<0.001** ^a^	Ref.	
Nuclear	124(80.0)	31(20.0)		3.42(2.14–5.45)	**2.74(1.67–4.51)**
**Marital status**					
Non-widowed	155(61.5)	97(38.5)	**0.162** ^a^	Ref.	
Widowed	100(68.5)	46(31.5)		1.36(0.88–2.09)	1.01(0.55–1.86)
**Number of children**					
>3	235 (62.3)	142 (37.7)	**0.002** ^b^	Ref.	
0–3	20 (95.2)	1 (4.8)		12.08(1.60–91.02)	
**Living arrangement**					
Family members	222(61.3)	140(38.7)	**<0.001** ^b^	Ref.	
Without family members	33(91.7)	3 (8.3)		6.93(2.08–23.04)	
**Present Income**					
No	37(62.7)	22(37.3)	0.814 ^a^	Ref.	
Yes	218(64.3)	121 (35.7)		1.07 (0.60–1.89)	
**Current habit of tobacco consumption**					
No	132(60.3)	87(39.7)	**0.081** ^a^	Ref.	
Yes	123 (68.7)	56 (31.3)		1.44(0.95–2.19)	1.23(0.77–1.97)
**Current habit of alcoholism**					
No	178 (62.7)	106 (37.3)	0.360 ^a^	Ref.	
Yes	77 (67.5)	37 (32.5)		1.23(0.78–1.96)	
**Suffered from disease**					
No	71 (60.7)	46 (39.3)	0.364 ^a^	Ref.	
Yes	184 (65.5)	97 (34.5)		1.22(0.78–1.91)	
**Socio-economic status**					
Upper Middle	23 (38.3)	37 (61.7)	**<0.001** ^a^	Ref.	
Lower Middle	51 (56.7)	39 (43.3)		2.10(1.08–4.09)	1.98(0.98–4.02)
Upper Lower	106(66.7)	53(33.33)		3.21(1.73–5.95)	**2.72(1.35–5.48)**
Lower	75(84.3)	14 (15.7)		8.61(3.9818.65)	**5.42(2.28–12.92)**

^a^ = Pearson chi- square p- value

^b^ = Fischer’s exact test p- value

Ref.: Reference, Cat: categories Variables included in the adjusted models—Age, Sex, Types of family, Education, Marital status, Tobacco consumption, Socio-economic status

### Result of qualitative study

A qualitative study was conducted to investigate perceptions of elder abuse and uncover underlying factors, as seen through the eyes of social leaders, local government representatives, and elderly individuals themselves. Various aspects and dimensions of elder abuse were collected from participants. It presents the codes, themes, and subthemes that have emerged from the study.

#### Theme: Perception of elder abuse

*Subtheme*: *Mistreatment by juniors and family members*. Participants shared a distressing experience of mistreatment by juniors and family members. One participant highlighted issues like communication difficulties, inadequate care and threat of eviction.

One elderly woman shared her experience, with tearful eyes, stating *"I am currently living alone and my son and daughter-in-law reside nearby*. *Whenever I receive my allowance*, *they come and demand to give money to them*. *However*, *I usually don’t comply with their requests*. *Since the day my husband passed*, *I’ve been living alone*. *Despite living right in front of my house*, *they eat their meals without inviting me or asking if I need anything*.*”*

*Vulnerability and negligence*. Respondents, including the local government representative and member of a Jestha nagarik sangh Sindhuli (Senior Citizens Association Sindhuli) shared a common understanding that growing older can make daily tasks harder due to reduced physical strength. Consequently, elderly parents might get susceptible of being neglected, and might be overlooked by younger family members. During the FGD sessions, participants noted that some people think elderly individuals are incapable of tasks due to age, and even consider death better than inactivity.

One participant shared a troubling incident from their village regarding the abuse from family members. *They explained*, *"In our village*, *there was an elderly woman from poor family who fell sick*. *To support her*, *all the members of our village collected funds and handed them over to her son for her medical treatment*. *Sadly*, *the son misused the entire fund by spending it on gambling and card games instead of using it for his mother’s treatment*.*"*

The qualitative study demonstrates a shared understanding among participants, including the *local official representative*, that ageing can make daily tasks harder due to reduced physical strength. This can lead to neglect of elderly parents, who might be overlooked in favor of younger family members. This perspective aligns with the quantitative findings, in which almost half of the respondents (46%) reported experiencing neglect from their family members.

#### Theme: Reason behind elder abuse

*Subtheme*: *Economic factors*. According to the respondents view, poverty as a significant contributor to the occurrence of elder abuse. Limited access to essential resources, notably food and basic necessities, due to economic constraints emerges as a key factor making the elderly more susceptible to abuse.

These qualitative perspectives correlated with the quantitative findings, where a strong link between socio-economic status and the prevalence of elder abuse was revealed. The respondents’ perspectives align with the quantitative evidence that individuals from lower socio-economic status face a high risk of abuse compared to those with higher socio-economic status.

*Subtheme*: *Family dynamics*. The family dynamics surrounding elder abuse shows a complex interplay of responsibilities and challenges. Respondents mentioned that sons, daughters, and in-laws sometimes think about abandoning their elderly parents when they perceive them as burdens. This happens when they believe they don’t have enough time to care for them. As an alternative, they place their elderly parents in homes where they can get regular meals and better care.

The triangulated findings demonstrate how family dynamics relate to elder abuse. Similarly finding was revealed from the quantitative study where a connection was found between elder abuse and belonging to a nuclear family.

One participant shared an emotional experience of loneliness despite having children. She expressed, *" I find myself living alone*. *Although I have a son and a daughter*, *I still experience profound loneliness*. *This feeling weighs heavily on me*.*"*

#### Theme: Barriers to reporting elder abuse

*Subthemes*: *Emotional attachment and love for family*. Some participants from FGD sessions shed light on the profound emotional attachment and love that many elderly individuals hold for their children, despite experiencing abuse.

One participant expressed *"Many of us cannot report the abuse because we still love our children despite their actions*.*"*

*Subtheme*: *Fear of disturbing home environment*. Fear plays a significant role in preventing the reporting of elder abuse, as highlighted by most of the Participant. Elderly individuals often fear that reporting such cases may disrupt the harmony within their homes. The desire to maintain a peaceful family environment, even in the face of mistreatment, contributes to the silence surrounding elder abuse.

## Discussion

This study assessed the prevalence of abuse and its associated factors among elderly population of Kamalamai Municipality of Sindhuli district, Nepal. In this study, the prevalence of abuse among the elderly population was found to be 64.1%. This finding is consistent with previous studies conducted across various regions of Nepal. A study by Bhandari et al. in Pokhara, Nepal in 2020 reported a prevalence of 65.6% among the elderly who had experienced some form of abuse [11]. In the context of Asian countries, Chandanshive et al. found that approximately (19.4%) older adults in India reported experiencing some form of abuse [16]. Similarly, a study in Singapore conducted by S. Chokkanathan in 2018 reported a prevalence rate of elder abuse at 8.3% [17].

In this study, we observed distinct patterns in the prevalence of elder abuse both within and beyond Nepal. Within Nepal, our findings align somewhat closely with studies conducted in different Nepali communities, suggesting shared cultural norms and socioeconomic conditions that may influence the prevalence rates. Additionally, most elderly individuals within the Nepali community are either left alone or with their daughters-in-law and grandchildren. However, when looking beyond Nepal’s borders, significant disparities become evident. Elder abuse rates in Kamalamai Municipality, Sindhuli district, Nepal appear notably higher than those reported in an urban slum of eastern India and Singapore, emphasizing potential cross-cultural variations in how elder abuse is understood and addressed.

This study identified the prevalence of various types of elder abuse among participants, with neglect (46.0%) and psychological abuse (42.5%) being the most common forms, followed by a financial abuse (24.4%), physical abuse (4.8%). In contrast, a study conducted in India found that 19.4% of older adults faced at least one form of abuse in the past year, with psychological abuse reported high at (11.1%), financial abuse at (4.2%) and physical abuse at (3.3%) [16]. The higher prevalence of neglect and psychological abuse in our study may reflect the socio-cultural context of Nepal, where changing family dynamics could lead to increases it.

In this study, it shows a significant association between family types and the prevalence of elder abuse, with an Adjusted Odds Ratio (AOR) of 3.42. Specifically, our findings suggest that living in a nuclear family setting increases the likelihood of experiencing elder abuse. This observation aligns with a study conducted by Wolde et al. in Ethiopia in 2022, which also reported a significant relationship between family size and elder abuse [18]. One possible reason for this trend is the adjustment difficulties and economic dependency experienced by the elderly. It is likely that elderly individuals living in nuclear families are more vulnerable to neglect, as they rely heavily on their family members for support. However, caregivers in such situations may face the challenge of balancing care for their aging parents with other responsibilities, potentially leading to an increased risk of elder abuse.

This study demonstrates a clear statistical association between socioeconomic status and the prevalence of abuse among older adults. Findings indicate that individuals with lower socioeconomic statuses are significantly more vulnerable to experiencing abuse compared to those with higher socioeconomic statuses. This observation finds support in a study conducted in Iran, which similarly identifies a substantial link between socioeconomic inequality and elder abuse, with a higher prevalence of abuse among economically disadvantaged elders [19]. Furthermore, a study conducted in India in 2021 further corroborates our findings, revealing that older adults from extremely low-income families are at a heightened risk of abuse [20]. A study in Nepal also shows that socioeconomic status is closely linked to abuse [14].

The implication of these findings are profound. This association between lower socioeconomic status and increased risk of elder abuse highlights the need for targeted interventions and support systems for economically vulnerable elderly populations. It suggests that policies aimed at improving the economic conditions of older adults could potentially mitigate their risk of abuse.

The majority of the participants in the focus group discussions, key informant interviews, and in-depth interviews expressed their views on perception of abuse, reasoning, vulnerability by age, reported cases, and personal experiences. They also mention about the barriers to reporting abuse. Furthermore, participants shared their perspectives on past and present conditions, along with their recommendations on each topic they discussed. In a study conducted in Sweden on the experience of elder abuse, several subcategories emerged from the respondents. These subcategories included vulnerability in old age, experiences from earlier life, perception of abuse, and consequences and suffering from abuse. When recounting experiences from earlier life, respondents in the Swedish study shared stories of the challenges they had faced, the support they received, and their individual strengths and vulnerabilities [21]. In this study, under the categories of personal experiences and reported cases, respondents brought attention to additional facets of elder abuse, such as financial exploitation, food withholding, loneliness, and physical abuse.

These variations in perspectives between the two studies may reflect cultural or contextual differences. This highlights the importance of considering local and cultural variations when addressing elder abuse comprehensively.

In this study, participants consistently highlighted financial exploitation and neglect as prevalent forms of elder abuse. They draw attention to distressing instances like pressuring older individuals into signing away their property and assets, as well as withholding necessary treatment from elderly parents. Our findings resonate with another study conducted in Ghana, which focused on the knowledge of health workers regarding elder abuse and neglect. This study uncovered a distressing pattern wherein some family members neglect their elderly relatives to evade the responsibilities that arise when an older adult is admitted to a care facility. Such neglect often revolves around avoiding financial obligations, including fees and medication costs. Alarmingly, certain relatives even make the heartbreaking decision to abandon their elderly family members upon admission [22]. These parallel findings from this study and the study conducted in Ghana underscore the global significance of elder abuse, which transcends geographic boundaries. This emphasize the critical need for comprehensive efforts to address these issues on a global scale, highlighting the importance of ongoing research and policy initiatives aimed at safeguarding the well-being and dignity of older adults worldwide.

### Strength

This study used both qualitative and quantitative methods, which provides a comprehensive and multidimensional perspective on elder abuse. The use of a structured validated tool enhanced the reliability and validity of the data. Conducting the research in a community setting allowed for a deeper exploration of the issues and challenges faced by the elderly population in their natural environment, which enriched the context and applicability of the findings.

### Limitations

The study was conducted in a semi-urban area, the findings may not be fully generalizable to either highly urbanized area or remote rural regions. Since this study involved elderly participants, some may have had difficulty accurately recalling events from the past year, leading to potential recall bias.

## Conclusion

This study highlighted that a significant proportion (64.1%) of elderly participants in Kamalamai Municipality experienced abuse, with neglect and psychological abuse being the most prevalent forms. The findings showed that multifaceted nature of elder abuse, including the perceptions, reasons, vulnerabilities, personal experiences and the barriers to reporting it.

The study revealed that elders living in nuclear families and those from lower socio-economic background were significantly more vulnerable to abuse. To address this issue, it is essential to maintain and reestablish joint family systems, which provide better care and security for the elderly, and to implement targeted measures to improve socio-economic conditions. These measures could include ensuring access to healthcare and facilitating income -generating opportunities for elderly individuals who are able and willing to work as well as for their families. Furthermore, addressing elder abuse requires the development of comprehensive educational programs for the elderly, their families, caregivers, and the communities. These programs should focus on recognizing signs of abuse and promoting respect for the rights and dignity of the older individuals. Moreover, it is essential to encourage reporting of any form of elder abuse to higher authorities through proper channels. Further research on elder abuse in different areas of Nepal will help to understand better and create more tailored solutions to ensure the dignity and well-being of the elderly population.

## Supporting information

S1 Questionnaire (DOCX)

S1 Data (XLSX)
