# Supplementary material for: Abuse and its associated factors among elderly population of Kamalamai Municipality of Sindhuli District, Nepal
Source: PLoS One. 2025 Jan 24;20(1):e0316078. doi: 10.1371/journal.pone.0316078 (PMC11760561; doi:10.1371/journal.pone.0316078)
Supplement: S1 Questionnaire — (DOCX) [file pone.0316078.s001.docx]

# ANNEXURE I

# English Questionnaire for data collection

**SCHOOL OF PUBLIC HEALTH AND COMMUNITY MEDICINE BP KOIRALA INSTITUTE OF HEALTH SCIENCES, DHARAN, NEPAL**

Greeting! My name is Jwala Subedi. Under the guidance of my guide Additional Professor Dr.Deepak Kumar Yadav , I am doing research on **“ABUSE AND ITS ASSOCIATED FACTORS AMONG ELDERLY POPULATION OF KAMALAMAI MUNICIPALITY OF SINDHULI DISTRICT”.** I assure you the data taken from you will be solely used for academic purpose only. If you wish to take part in this survey. I will ask you question related to the research.

**Participant identification number for the study:**

Respondent’s signature _____________

Researcher’s signature ______________ Date:

Right Left

SectionA:

Socio-demographic information

| **SN** | **Questions** |
| --- | --- |
| **A1** | Age of Participants…………….. Ward – |
| **A2** | Sex  1.Male 2.Female 3. Other |
| **A3** | Ethnicity  1.Brahmin/Cheetri 2. Dalit 3.Janajati 4.Madhesi 5.Muslim 6. Others |
| **A4** | Religion   1. Hindu 2. Boudha 3. Christian 4. Islam 5. Kirat 6. Others |
| **A5** | Type of Family  1.Nuclear 2.Joint 3. Extended |
| **A6** | Number of Family Members: …….. |
| **A7** | Educational Status  1.illiterate 2.informal education 3.Basic level (class 1-8)  4. Secondary level (class 9-12) 5. Graduate  6.Masters or above |
| **A8** | Marital Status  1.Unmarried 2.Married 3. Divorced  4.Widow 5.Widower |
| **A9** | Housing Condition  1.Owner 2.Rented |
| **A10** | Occupation  1.Unemployed 2.House maker 3. Agriculture  4.Government Services 5. Retired 6. Non-government services 7. Other |
| **A11** | Number of Children  1.1-3 2.4-6 3.more than 7 |
| **A12** | With whom you are living now?  1.Son 2.Daughter 3. Daughter in law 4.Husband  5.Wife 6.Others |
| **A13** | Own source of income :   1. Yes 2. No ( if no , go to section B) |
| **A 14** | If yes, Present source of monthly income   1. Pension 3. Savings 2. Old age allowance 4. Business 5. Others…. (specify) |
| **A 15** | Your Income per month NRS……………… |

**Section 2 : Socio economic status**

**Education Status of Head of household:**

| **Education** | **Score** |
| --- | --- |
| Professional or honors | 7 |
| Graduate or postgraduate | 6 |
| Intermediate of post high school diploma | 5 |
| High school certificate | 4 |
| Middle school certificate | 3 |
| Primary school or literate | 2 |
| Illiterate | 1 |

**Occupation of Head of household:**

| **Occupation** | **Score** |
| --- | --- |
| Profession | 10 |
| Semi-profession | 6 |
| Clerical, shop owner, farmer | 5 |
| Skilled worker | 4 |
| Semi- skilled worker | 3 |
| Unskilled worker | 2 |
| Umemployment | 1 |

**Family income per month**

| **NPR** | **Score** |
| --- | --- |
| ≥97451 | 12 |
| 48751- 97450 | 10 |
| 36551-48750 | 6 |
| 24351-36550 | 4 |
| 14551-24350 | 3 |
| 4851-14550 | 2 |
| ≤4850 | 1 |

Section C: Behavior and health related questions

| **B1** | Do you have habit of smoking and chewing tobacco?  1.Yes 0.No |
| --- | --- |
| **B2** | Do you have habit of drinking alcohol?  1.Yes 0.No |
| **B3** | Have you suffered from any illness in last year? **(If no go to Q.No. B5)**  1.Yes 0.No |
| **B4** | If yes, what type of health problems did you face?  1.Hypertension 2.Diabetes 3. Cancer 4. Tuberculosis  5.Mental illness 6.Disabled 7. Others |
| **B5** | What difficulties did you face due to health problems?  1.Difficulty in daily living activities 2.Couldn’t eat favorite food  3. Couldn’t go anywhere 4.Others |

Section C: Abuse related questions Physical Abuse

| **C1** | Have you been force to work at come?  1.Yes 0.No |
| --- | --- |
| **C2** | Have you been struck, slapped or kick?  1.Yes 0.No |
| **C3** | Have you been tied down or locked in room?  1.Yes 0.No |
| **C4** | Has anyone touched you without your permission? |

|  | 1.Yes 0.No |
| --- | --- |
| **C5** | Have you had an object thrown at you?  1.Yes 0.No |
| **C6** | Have you been a victim of an attempted or completed  physical attack that was serious in nature? **(If no go to Q.No. D1)**  1.Yes 0.No |
| **C7** | If yes, what happened?  1.Hit 2.Slapped 3. Injured with weapon 4. Injured with instrument or object 5.Others |

Psychological or Emotional abuse

| **D1** | Are you satisfied with your life?  1.Yes 0.No |
| --- | --- |
| **D2** | If not, why?  1.Lack of due respect 2.Lack of support from family members  3. Others |
| **D3** | Do you ever feel alone?  1.Yes 0.No |
| **D4** | Have you been upset because someone talked to you in a way that made you feel shamed?  1.Yes 0.No |
| **D5** | Have you been threatened with punishment, deprivation or institutionalization?  1.Yes 0.No |
| **D6** | Have you been afraid of with your family? 1.Yes  0.No |
| **D7** | Have you received the silent treatment? |

|  | 1.Yes 0.No |
| --- | --- |
| **D8** | Have you been force fed?  1.Yes 0.No |
| **D9** | Has anyone called you unkind names or put you down?  1.Yes 0.No |
| **D10** | Has anyone made you feel small, such as treating you as a child?  1.Yes 0.No |
| **D11** | Has anyone behaved in ways that frighten or scare you?  1.Yes 0.No |
| **D12** | Has anyone not let you speak for yourself?  1.Yes 0.No |
| **D13** | Has anyone treated you in an undignified or inappropriate manner when assisting you with dressing, eating, bathing and so on?  1.Yes 0.No |
| **D14** | Has anyone kept things from you or lied about things you should know about?  1.Yes 0.No |

Neglect

| **E1** | Have you been left alone for long time?  1.Yes 0.No |
| --- | --- |
| **E2** | Do you lack needed medications or medical equipments such as eyeglasses, hearing aids, dentures, walkers?  1.Yes 0.No |
| **E3** | When you are in problem who will help you?  1.None 2.Society people 3.Family members 4.Others |

| **E4** | Do you have inadequate clothing?  1.Yes 0.No |
| --- | --- |
| **E5** | Do you have poor personal hygiene? **(**As evidenced by a noticeable odor, long and dirty fingernails, etc.)  1.Yes 0.No |
| **E6** | Do you lack access to needed areas of the home (bathtub, sinks, bed)?  1.Yes 0.No |
| **E7** | Do you lack sufficient care from your caregiver?  1.Yes 0.No |
| **E8** | Does your caregiver ignore signs and symptoms of your disease?  1.Yes 0.No |

Financial abuse

| **F1** | Does your caregiver depend on you for shelter or financial support?  1.Yes 0.No |
| --- | --- |
| **F2** | Has money been stolen from you?  1.Yes 0.No |
| **F3** | Have you been forced to sign financial or legal documents against your will and without your understanding?  1.Yes 0.No |
| **F4** | Has someone obtained money, property, or financial resources from you through cheating  1.Yes 0.No |
| **F5** | Has someone borrowed or taken money from you and not paid it back?  1.Yes 0.No |
| **F6** | Have any of your properties been sold without your permission?  1.Yes 0.No |

Sexual Abuse

| **G1** | Have you been forced into any sexual activities involving: observing sexual activities, touching?  1.Yes 0.No |
| --- | --- |
| **G2** | Have you ever been experienced sexual harassment, inappropriate flirting and indecent exposure?  1.Yes 0.No |
| **G3** | Have you been forced to use or listen to sexual language?  1.Yes 0.No |
| **G4** | Have you been sexually exploited (made to have sex for goods/money)?  1.Yes 0.No |

**FGD Guidelines**

| **Topic** | **Key questions** |
| --- | --- |
| Introduction | Can you describe what you will define as abuse among elderly? |
| Your experiences of elder abuse | *physical abuse, psychological abuse, financial abuse, sexual abuse and neglect,* can you describe your experience of elder abuse and neglect? |
| Communication of elder abuse | Can you describe how you get knowledge about situations of elder abuse?  What do you think are barriers and enablers to report elder abuse? |
| How to follow up on elder abuse | When you get knowledge about situations of elder abuse and neglect, how do you follow it up?  What do you do to prevent it from happening again? |
| Closure | Do you have anything to add that has not been mentioned?  How did you experience participating in this focus group? |

KII Guideline:

What do you understand by term elder abuse?

What do you think the causes of Elder abuse?

Is there any effect on health in the presence of abuse among elderly people?

Have you seen any kind of elder abuse in your family or neighborhood?

What are the consequences of elder abuse to the elder people and their family members?

What are your responsibilities to prevent abuse among elder people?

What are the responsibilities of palika or ward level to prevent abuse among elderly? Have they done any kind of activities to prevent it?
